# Pulmonary tumor embolism secondary to urothelial carcinoma of urinary bladder: case report and literature review

**DOI:** 10.1186/s43044-023-00422-w

**Published:** 2023-11-27

**Authors:** Abderrahmane Bouchaala, Ihssane Khalek, Oualid Kerrouani, Najat Mouine, Zouhair Lakhal, Aatif Benyass

**Affiliations:** 1 Clinical Cardiology Department, Cardiology Center, Mohammed V Military Instruction Hospital of Rabat, Mohammed V University, Mohammed Belarabi Elalaoui Av., B.P.6203, 10000 Rabat, Morocco; 2 Cardiac Intensive Care Unit, Cardiology Center, Mohammed V Military Instruction Hospital of Rabat, Mohammed V University, Rabat, Morocco; 3 Head of Cardiology Center, Mohammed V Military Instruction Hospital of Rabat, Mohammed V University, Rabat, Morocco

**Keywords:** Pulmonary embolism, Tumor thrombus, Urothelial carcinoma, Urinary bladder

## Abstract

**Background:**

Tumor embolism is the least well-described cause of pulmonary embolism, taking into account the non-specificity of radiographic and nuclear imaging results, the necessity of anatomopathological evidence and the frequency of deep venous thrombosis in the course of solid tumors, suggesting thus thromboembolism.

**Case presentation:**

We report a rare case of urothelial carcinoma of the urinary bladder associated with persistent pulmonary embolism despite being on different anticoagulation regimens, the patient was ultimately found to have tumor thrombus in the pulmonary trunk secondary to tumoral extension. We provide a literature review as well about the diagnosis, evaluation and prognosis and of this rare clinical entity.

**Conclusions:**

Our case highlights the importance of keeping this unusual etiology in mind, particularly when faced with pulmonary embolism occurring in the context of a solid tumor and the ineffectiveness of various anticoagulation protocols. Furthermore, it emphasizes the pivotal role of cytology in confirming diagnosis and guiding therapy.

## Background

Patients with cancer may present dyspnea at different phases of their disease evolution, with causes varying considerably according to the etiopathogenic mechanism, such as, but not limited to, pulmonary metastases, infection, anemia, thromboembolism, cardiac-associated comorbidities, pleural and pericardial effusions. Pulmonary tumor embolism represents an infrequent neoplastic complication characterized by the occlusion of pulmonary microvasculature by metastatic cellular aggregates. It results in a subacute and incremental onset of dyspnea, pulmonary hypertension, and, on occasion, culminates in sudden death [1].

While our current understanding of pathological mechanisms underlying tumor embolism remains poorly elucidated, the main evoked hypotheses are the diffuse vascular remodeling, a consequence of the dysregulation of signaling pathways that typically respond to the presence of an embolic cell or other intravascular injury. The other mechanism is the increase in pulmonary vascular resistance via mechanical occlusion of the pulmonary vascular bed [1, 2]. The antemortem diagnosis is rarely recognized due to the requirement of a pathological evidence and short-term poor prognosis. In this report, we present a rare case of pulmonary tumor embolism secondary to urothelial carcinoma of the urinary bladder diagnosed by pulmonary cytology.

## Case presentation

**Fig. 1:**
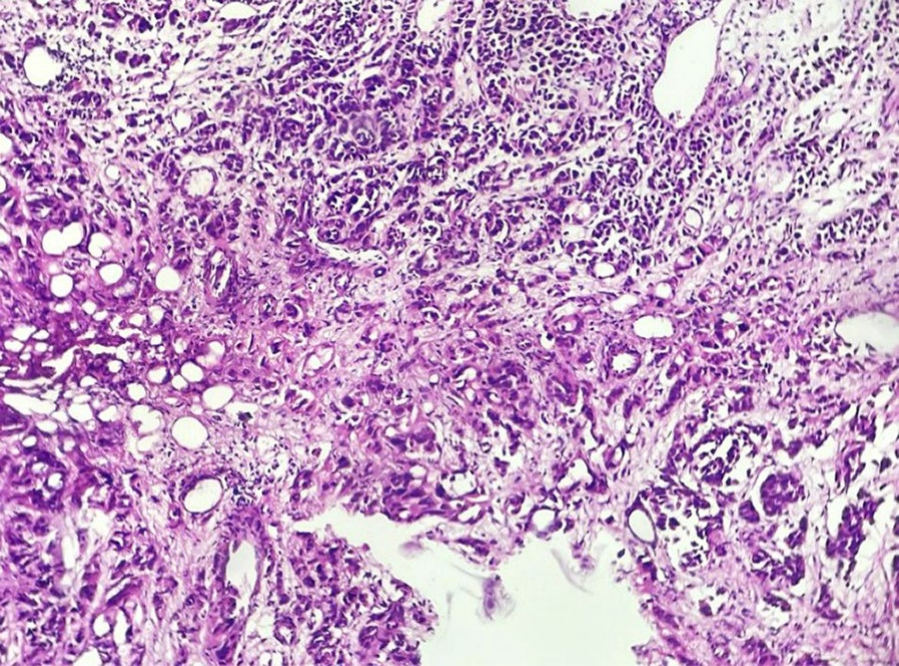
Histopathology of high-grade urothelial carcinoma of urinary bladder: patches of tumor cells showing complex papillary architecture and loss of polarity. Hematoxylin and eosin H&E staining (magnification × 20)

A 57-year-old female patient with no notable medical history who presented with acute dyspnea. One month prior to this presentation, she was diagnosed with bladder tumor revealed by macroscopic painless hematuria. Pathological assessment confirmed the presence of urothelial carcinoma with infiltration into the muscular layer (Fig. 1). Initially admitted to the urological surgery department for therapeutic management, the patient subsequently developed acute dyspnea and was transferred to the intensive care unit. On admission, the patient exhibited (respiratory rate of 37 cycles per minute), tachycardia (heart rate of 135 beats per minute) and normotensive blood pressure (123/67 mmHg). Peripheral oxygen saturation (SpO2) measured at 94%. There were no signs of congestive heart failure, and cardiopulmonary auscultation was unremarkable. Peripheral pulses were palpable and symmetrical. The physical examination of the other systems, notably the neurological, pleuropulmonary and digestive systems, was unremarkable, and there was no evidence of active hematuria observed in the urinary output. Blood gas analysis showed a mild hypocapnia. Laboratory assessment showed anemia (hemoglobin level of 9 g/dL) and thrombocytopenia (12 × 104 /µL), while liver and renal functions remained within normal ranges (Table 1).

**Table 1:** Biological parameters on admission to emergency room

Variable	Results
Sodium (mmol/L)	141
Potassium (mmol/L)	4.3
Chloride (mmol/L)	99
Blood glucose (g/L)	1.20
Serum bicarbonate (mmol/L)	27
Urea (g/L)	0.31
Creatinine (mg/L)	10.7
ASAT (U/L)	14
ALAT (U/L)	17
GGT (U/L)	25
pH	7.44
pO2 (mmHg)	89
pCO2 (mmHg)	33
CRP (mg/dL)	5.5
Procalcitonin (ng/mL)	0.07
White blood cells (/mcL)	9500
Neutrophils	5400
Hemoglobin (g/dL)	9
Platelets (/mcL)	120000

**Fig. 2:**
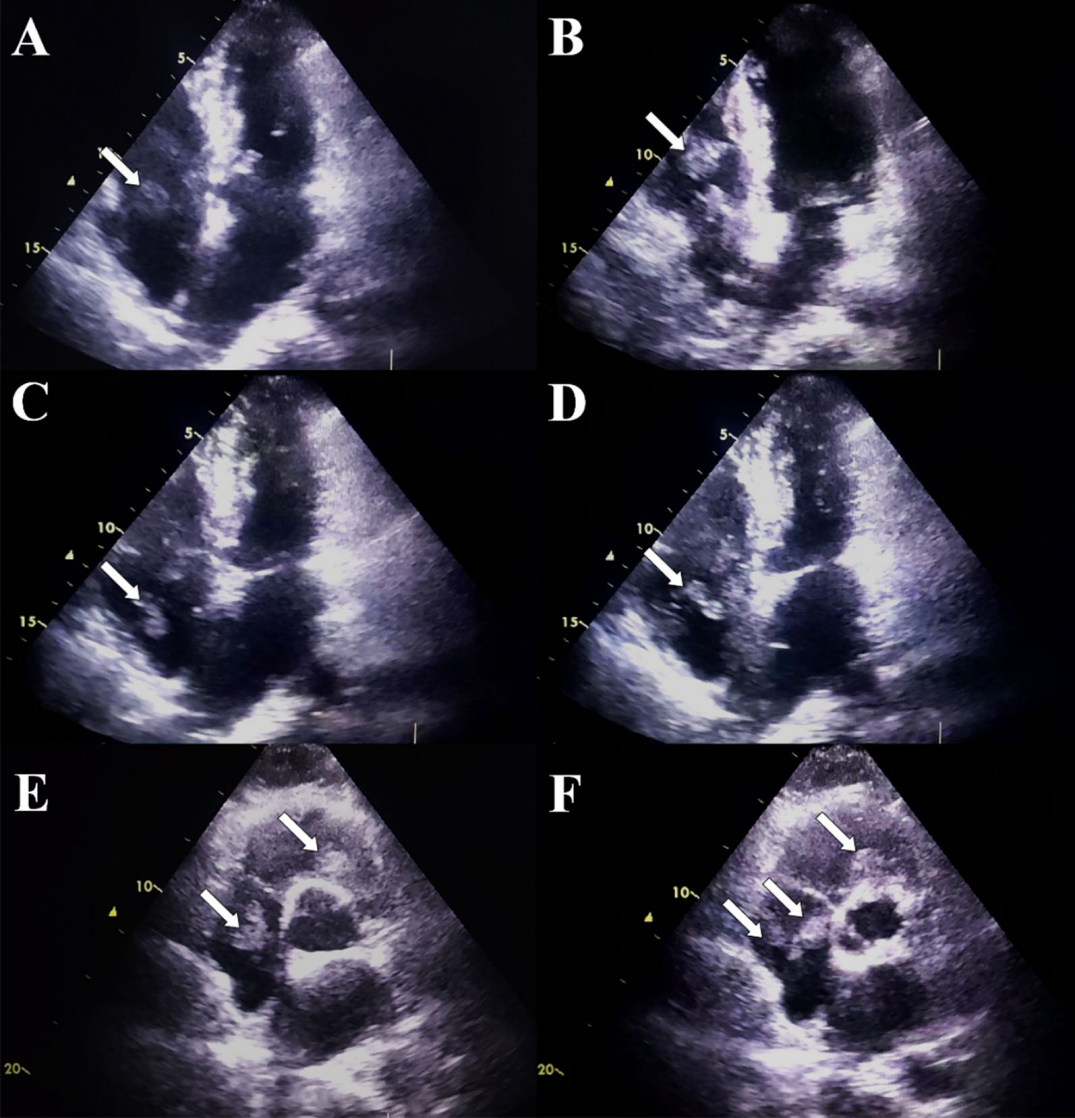
Transthoracic echocardiography of the patient in the apical four-chamber view (**A**, **B**, **C** and **D**) and the parasternal short axis view (**E** and **F**) in diastole (**A**, **B** and **E**) and systole (**C**, **D** and **F**) showing the presence of several centimetric emboli floating in the right atrium and ventricle (white arrows)

**Fig. 3:**
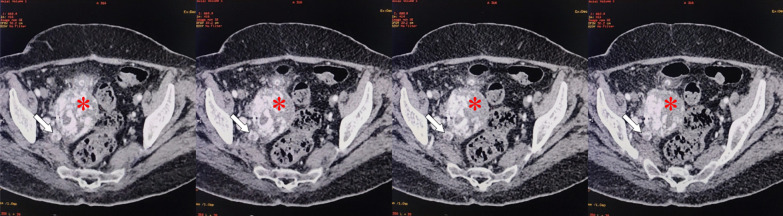
Abdominal CT axial scans showing the urinary bladder tumor (red star) extending into the right internal iliac vein (white arrow)

**Fig. 4:**
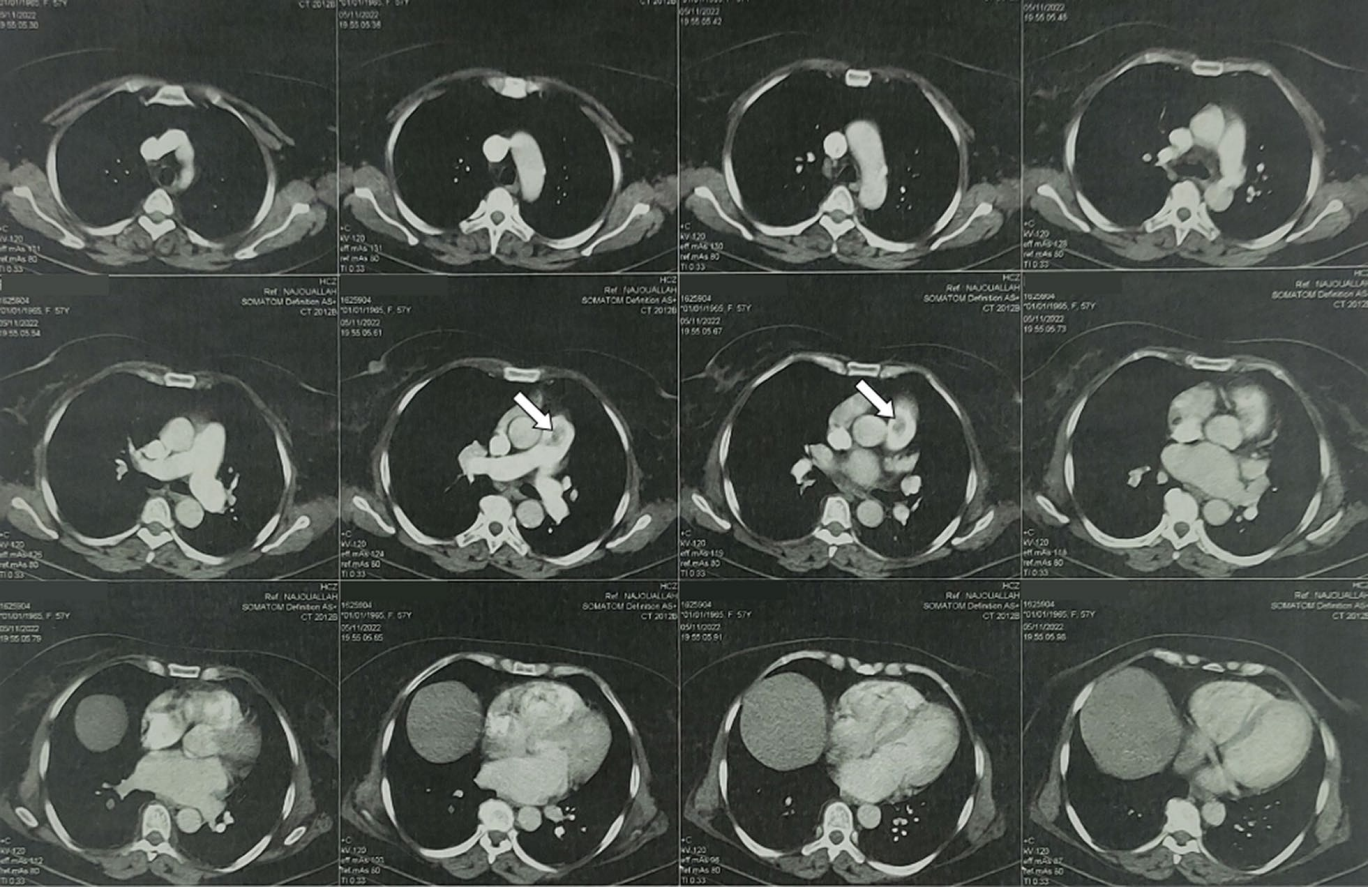
CT pulmonary angiogram showing proximal pulmonary embolism (white arrows)

Transthoracic echocardiography revealed a hyperkinetic right ventricle, mild tricuspid regurgitation and the presence of multiple hypermobile echogenic elements, varying in size, with spherical and oval shapes. These elements were observed floating within the right heart chambers and pulmonary trunk (Fig. 2), and systolic pulmonary artery pressure was estimated at 30 mmHg. Left systolic function was preserved, and filling pressures were normal. Abdominal CT scan revealed the presence of tumoral invasion of the bladder tumor into the right internal iliac vein (Fig. 3), and chest CT angiography showed multiple pulmonary emboli in main pulmonary arteries, with no other metastatic localizations (Fig. 4).

On the therapeutic level, thrombolytic therapy was deferred due to the hemodynamic stability, and the patient was considered at intermediate risk level. A variety of anticoagulant regimens were employed, with the initial use of the direct oral anticoagulant (DOAC) Apixaban at a dose of 10 mg b.i.d during the initial week, followed by a reduced dose of 5 mg b.i.d. for three weeks. Subsequent echocardiographic assessments revealed no regression of pulmonary or right heart emboli, prompting a transition to an Enoxaparin regimen of 80 mg every 12 h during the next four weeks. In the absence of observable regression on ultrasound examination, Rivaroxaban was introduced at a dosage of 15 mg b.i.d. To assess the impact of these emboli on pulmonary vasculature and gauge the extent of pulmonary hypertension, a right heart catheterization was conducted with aspiration cytology, revealing the presence of clusters of large atypical cells resembling urothelial carcinoma cells.

With the confirmed diagnosis of pulmonary tumor embolism secondary to urothelial carcinoma, patient initiated palliative chemotherapy in conjunction with anticoagulation. Patient exhibited favorable clinical recovery and was transferred lately to urology department for further management of the primary tumor. However, three days after her transfer, the patient experienced acute respiratory distress requiring orotracheal intubation and mechanical ventilation. Unfortunately, her condition deteriorated, and she died on the fifth day.

## Discussion

Pulmonary tumor embolism is an uncommon complication of solid tumors; it has been sporadically reported, among others, in renal, testicular, breast, gastric and liver carcinomas. Among these reported cases, a smaller percentage of patients have documented morbimortality directly attributable to the embolism, which makes its nosological assessment challenging compared to other forms of pulmonary embolism [1, 3, 4]. Taking account of these difficulties, antemortem prevalence is poorly established in the literature; nonetheless, autopsy series provided an epidemiological estimation, indicating that the incidence of tumor embolism among patient with solid malignancies ranged between 1% and 2.4% [5, 6].

Tumor embolism should be discussed in a cancer patient who has presented with acute dyspnea, respiratory distress or clinical signs of pulmonary hypertension. In our case, early suspicion has been triggered by tumoral invasion of internal iliac vein and the persistence of pulmonary embolism in spite of various anticoagulation regimens. The observation of the resistance to anticoagulant therapy or the development of new emboli during treatment has been noted in previous reports of tumor embolism. These factors should be regarded as major clues in distinguishing between thromboembolism and tumor embolism [2, 3].

To date, the contribution of medical imaging to diagnosis remains limited; although CT scans coupled with angiography can be used to diagnose proximal embolism with no major difference to thromboembolism, a retrospective study showed multifocal beading and dilatation of peripheral pulmonary arteries, principally in a subsegmental distribution and involving multiple lobes in patient with tumor emboli [7]. Lung ventilation-perfusion imaging has shown greater diagnostic utility, as perfusion defects caused by tumor emboli are characteristically multiple, symmetric, and more peripheral compared to thromboembolism defects [8].

The gold standard for diagnosis is based on pathological findings; the use of right heart catheterization and pulmonary vasculature cytology can confirm the tumoral nature of thrombus. In our case, the abundance of thrombi in the right cavities, pulmonary trunk and its branches facilitated the sampling process. Surgical biopsy is mostly based on pulmonary endarterectomy associated with thromboembolectomy; even if this procedure provides definite pathological diagnosis, it is associated with an increased operative risk, especially in these frail patients [3].

While treatment of thromboembolism occurring in patients with active tumors is well-established [9], therapeutic options for tumor embolism remain limited, taking into account the dual high risks, thrombotic and hemorrhagic. Therapy of the primary tumor is often considered the management cornerstone, including surgery, chemotherapy or chemo-radiotherapy. However, patient outcomes are primarily contingent on tumor extension, malignancy, and its receptivity to chemotherapy [2].

## Conclusions

Tumor embolism should be considered among the first diagnoses in patients with documented malignant tumors presenting with acute or subacute dyspnea, massive invasion of systemic venous return or the right heart chambers and persistence of pulmonary emboli despite efficient anticoagulation regimens. Nonetheless, owing to the scarcity of this condition, the non-specific findings in radiographic tests and the necessity for pathological confirmation, tumor embolism remains frequently undiagnosed.

## Data Availability

No datasets were generated or analyzed during the current study.

## Abbreviations

*SpO2*: Peripheral oxygen saturation
*CT scan*: Computed tomography scan
*DOAC*: Direct oral anticoagulant
